# Constructing a prognostic model for colon cancer: insights from immunity-related genes

**DOI:** 10.1186/s12885-024-12507-z

**Published:** 2024-06-24

**Authors:** Ansu Li, Qi Li, Chaoshan Wang, Xue Bao, Feng Sun, Xiaoping Qian, Wu Sun

**Affiliations:** 1 Department of Clinical Laboratory, Nanjing Drum Tower Hospital, The Affiliated Hospital of Nanjing University Medical School, Nanjing University, Nanjing, China; 2 Department of Oncology, Affiliated Hospital of Medical School, Nanjing Drum Tower Hospital, Nanjing University, Nanjing, China; 3 Department of Pathology, Affiliated Hospital of Medical School, Nanjing Drum Tower Hospital, Nanjing University, Nanjing, China; 4 Department of Cardiology, Nanjing Drum Tower Hospital, The Affiliated Hospital of Nanjing University Medical School, Nanjing, China; 5 Division of Gastric Surgery, Department of General Surgery, The Affiliated Hospital of Medical School, Nanjing Drum Tower Hospital, Nanjing University, Nanjing, China

**Keywords:** Colon cancer, RBM47, CXCL13, Tumor microenvironment, Tertiary lymphoid structure

## Abstract

**Background:**

Colon cancer (CC) is a malignancy associated with significant morbidity and mortality within the gastrointestinal tract. Recurrence and metastasis are the main factors affecting the prognosis of CC patients undergoing radical surgery; consequently, we attempted to determine the impact of immunity-related genes.

**Result:**

We constructed a CC risk model based on ZG16, MPC1, RBM47, SMOX, CPM and DNASE1L3. Consistently, we found that a significant association was found between the expression of most characteristic genes and tumor mutation burden (TMB), microsatellite instability (MSI) and neoantigen (NEO). Additionally, a notable decrease in RBM47 expression was observed in CC tissues compared with that in normal tissues. Moreover, RBM47 expression was correlated with clinicopathological characteristics and improved disease-free survival (DFS) and overall survival (OS) among patients with CC. Lastly, immunohistochemistry and co-immunofluorescence staining revealed a clear positive correlation between RBM47 and CXCL13 in mature tertiary lymphoid structures (TLS) region.

**Conclusion:**

We conclude that RBM47 was identified as a prognostic-related gene, which was of great significance to the prognosis evaluation of patients with CC and was correlated with CXCL13 in the TLS region.

**Supplementary Information:**

The online version contains supplementary material available at 10.1186/s12885-024-12507-z.

## Introduction

According to the American Cancer Society, approximately 106,970 individuals were diagnosed with CC in the United States in 2023 [1]. Despite advancements in diagnosis and therapy, the prognosis of CC remains unfavorable due to its high rates of metastasis and post-intervention recurrence [2]. Surgical removal does not guarantee a favorable outcome, as approximately 30–40% of patients with stages II and III CC develop recurrence, which is the primary cause of mortality associated with this disease [3]. Consequently, an urgent need exists to identify additional biomarkers for diagnosis and prognosis as well as therapeutic targets to improve long-term outcomes in patients with CC.

The tumor microenvironment (TME)—consisting of malignant cells, infiltrating immune cells, the extracellular matrix, resident mesenchymal cells and other constituents—plays a crucial role in influencing cancer progression and therapeutic outcomes [4]. Typically, the immune system serves as a defense mechanism against neoplasia development. However, mounting evidence suggests that immune cell [5, 6] infiltration is closely associated with the prognosis of CC patients. Specifically, the presence of cytotoxic CD8^+^ T cells within the central or peripheral regions of the tumor is indicative of a reduced risk of recurrence in patients with CC [7]. Additionally, constructing prognostic predictive models for patients with CC relies on the quantification of tumor-associated neutrophils (TANs) [8], regulatory T cells (Tregs) [9] and tumor-associated macrophages (TAMs) [10], rendering them more dependable than conventional indicators for prognostic evaluation. Furthermore, higher densities of tumor-infiltrating dendritic cells (DCs) have been linked to prolonged survival in individuals with CC, suggesting a novel prognostic determinant [11]. Moreover, a higher density of TLS was closely correlated with an improved prognosis in patients with colorectal cancer (CRC) [12]. Consequently, a better understanding of immune cell biology within the CC microenvironment facilitates the elucidation of potential tumorigenic mechanisms.

In the current investigation, using single-cell and bulk transcriptome sequencing analysis enabled the identification of six immune-related differentially expressed genes (DEGs) that exhibited a significant association with DFS and OS in the context of CC. Notably, our findings indicate a significant decrease in RBM47 expression in CC tissues compared with that in adjacent normal tissues. RBM47 primarily functions as an RNA-binding protein (RBP) and may potentially exert regulatory effects on DNA, thereby influencing gene regulatory functions [13]. Various cancer types can be affected by the aberrant expression of RBM47, which can affect transcriptional and post-transcriptional regulation. Furthermore, RBM47 exhibits tumor-suppressive effects in various solid tumors, including lung [14], breast [15], hepatocellular [16] and colorectal cancers [17]. However, it is worth noting that RBM47 may present malignant significance on nasopharyngeal carcinoma and non-small cell lung cancer survival. These findings indicate that RBM47 exerts double-edged functions in cancer development in a specific disease context. Nevertheless, it is unclear exactly how RBM47 contributes to cancer cell immunity and CC development.

In this context, we investigated the relationship between RBM47 expression levels and the clinicopathological features and prognosis of patients with CC. We also probed insight into TMB, MSI and neoantigen mediated by RBM47. Moreover, CXCL13 and RBM47 expression levels were correlated. Additionally, hematoxylin and eosin (H&E) and co-immunofluorescence showed RBM47 expression in TLS. Overall, this study enhances our knowledge of how the TME affects CC outcomes, providing valuable insights for clinical management and targeted therapy.

## Materials and methods

### Data sources and processing

RNA sequencing (RNA-seq) data, somatic mutation data and clinical information of 41 normal colon tissue samples and 448 CC tissue samples with survival information were obtained from TCGA database (TCGA-CC, https://portal.gdc.cancer.gov/). The scRNA-seq data for 10 normal colon tissue samples and 23 CC tissue samples from the GSE132465 dataset, the RNA-seq data for 566 CC tissue samples and 19 normal colon tissue samples from the GSE39582 dataset, and the survival data for 121 CC patients from the GSE41258 dataset were all obtained from the GEO database. (https://www.ncbi.nlm.nih.gov/).

### Patients

Tissue specimens from 118 patients with CC (Table 1) who underwent surgery between 2018 and 2020 were obtained from the Nanjing Drum Tower Hospital. All patients were followed up until January 2024. OS was calculated from the date of surgery until death or the date of the last follow-up. We calculated DFS from the date of surgery to the date of any diagnosis of progression. Fresh tumor tissue samples were obtained from 20 patients with CC during surgery at our hospital. Histopathological and clinical findings were scored according to the American Joint Committee on Cancer guidelines (8th Edition). The basic information and clinicopathological features of the patients are collected in Supplementary Table S1.

**Table 1:** Clinicopathological characteristics of involved colon cancer patients

Characteristics	RBM47 expression		*P value*
	Low (*N* = 59)	High (*N* = 59)	
Age(years)			0.87
Mean ± SD	60.2 ± 11.6	59.8 ± 13.9	
Gender			0.57
Male	37	34	
Female	22	25	
Differentiation			0.023
Poor	18	12	
Moderate	41	41	
Well	0	6	
T stage			0.016
T1	0	2	
T2	0	4	
T3	51	48	
T4	8	5	
N stage			0.0089
N0	15	30	
N1	31	24	
N2	13	5	
AJCC stage			0.0077
II	15	29	
III	44	30	
Location			0.85
Right	29	30	
Left	30	29	
MMR status			0.018
dMMR	4	13	
pMMR	55	46	
Chemotherapy			0.62
No	11	9	
Yes	48	50	

### Screening of differential immune cells

We analyzed the proportion of 22 immune cells in CC and normal colon tissue samples in GSE39582 and TCGA-CC datasets using the CIBERSORT algorithm and the differential immune cells between CC and normal colon tissue samples using the Wilcoxon test [18].

### Weighted gene co-expression network analysis (WGCNA)

Differential immune cell-associated module genes were screened using WGCNA [19] in all samples of the TCGA-CC datasets based on common differential immune cells in GSE39582 and TCGA-CC datasets. First, the Hclust function was employed to detect the presence of outlier samples, and then a feasible soft threshold β was selected. Then, by dynamic tree-cutting module identification, co-expression modules were obtained, hierarchical clustering trees were drawn, and modules with a correlation greater than 0.75 were merged by correlation analysis [20]. The relationships between each module and trait data were investigated using Pearson’s correlation analysis to identify the key module. The gene significance (GS, |GS|>0.2) and module membership (MM, |MM|>0.8) values of the modules were calculated, and the significantly-related module genes were screened [21].

### Analyzing the scRNA-seq data

The core cells were obtained by the “Seurat” R package [22]. The quality criteria were as follows: first, genes with a detection limit of three or fewer cells were excluded from the analysis. Additionally, cells with mitochondrial expression genes of less than 5% and low-quality cells with less than 200 genes were removed. The top 2000 highly variable genes with high intercellular coefficients of variation were screened using the Find Variable Features function. Principal component analysis (PCA) was performed on all samples in the scRNA data to minimize batch effects, with the top 50 principal components selected for overall dimensionality reduction analysis using the UMAP algorithm [23]. Marker genes were identified using FindAll Markers in the Seurat package. Several different cell types were annotated and visualized using a Single R package [24]. Cellular communication between different cell types was analyzed using the Cellchat R package [25]. DEGs were identified in the same type of immune cells between tumor and normal groups by “FindMarkers”.

### Establishment and evaluation of risk model

The “DESeq2” package version and the “ggplot2” plotting tool were used to evaluate and version DEGs between the normal and CC tumor groups in TCGA-CC with *p* < 0.05 and |Log2FC| > 1 [26]. Volcano and heatmaps were employed to visualize DEGs. Subsequently, Venn diagrams were utilized to visualize the immune cell-associated genes correlated with CC, and univariate Cox analysis was employed to select genes of significant value for patient survival based on their association with CC prognosis. Subsequently, the least absolute shrinkage and selection operator (LASSO) analysis was performed using the R package glmnet to screen for characteristic genes and construct prognostic risk models [27]. The prognostic model was structured using the following formula: Risk score = Coefgene1 × Expgene1 + Expgene2 × Coefgene2 + Coefgene3 × Expgene3 + …… + Coefgenen × Expgenen. TCGA-CC patients were divided into two risk categories (high- and low-risk category) based on the median risk score, and the difference in survival between the two categories was analyzed using Kaplan-Meier (KM) curves. The survROC package was deployed to plot ROC curves to assess the ability of the risk score to predict CC patient survival [28]. We then validated the feasibility of the prognostic model using the external validation GSE41258 dataset. The samples of patients with CC were grouped by optimal cut-off values for characteristic gene expression, and KM survival curves were plotted according to DFS outcome using the R package survivor. Patients with CC were grouped according to the best cut-off value of the CIBERSORT results, and KM survival curves for different key immune cell groupings were plotted using the R package survivor (OS and DFS).

### Assessment of the prognostic model

Clinicopathological features (T, N, M, age, sex and survival status) and risk scores were incorporated into chi-square tests to analyze the distribution and differences in the clinical characteristics between the two risk patient categories (high- and low-risk categories). Besides, a nomogram was constructed by the “rms” package combining clinicopathological characteristics with risk scores to predict the probability of survival at 1, 3 and 5 years for patients with CC in the TCGA-CC dataset [29]. The corresponding calibration and ROC curves were also drawn to assess the validity and dependability of the nomogram.

### Gene set enrichment analysis (GSEA) analysis

To confirm the biological features of hub genes, we counted their correlations with other genes and sorted them according to the results using a package called GSEA [30].

### Tumor mutation burden (TMB), microsatellite instability (MSI), neoantigen (NEO) and chemokines

TMB was calculated using somatic data from TCGA database as well as the MSI score. The relationship between hub gene expression and TMB or MSI was investigated using Spearman’s correlation analysis. The correlation between characteristic genes and chemokine-related genes was assessed using the cBioPortal and Tumor Immune Estimation Resource databases, respectively.

### Quantitative RT-PCR

RNA was extracted from the tumor and adjacent normal tissues using an RNeasy kit (Qiagen), and reverse transcription was carried out using a High-capacity cDNA Reverse Transcription kit (Applied Biosystems). For qRT-PCR, 10 ng of cDNA template was mixed with 1X SYBR Green PCR Master Mix (Applied Biosystems). The primer sequences are listed ((Supplementary Table S2). Amplification was optimized for all primers according to the following instructions. The 2 ^–ΔΔCT^ method was applied to normalize the expression of target genes to that of GAPDH.

### Immunohistochemistry (IHC)

Formalin-fixed, paraffin-embedded (FFPE) tissue sections underwent processes such as dewaxing, rehydration, and antigen retrieval, followed by blocking with secondary antibody source serum for both carcinoma and adjacent non-tumor sections. After blocking, the slides were incubated overnight with CXCL13 (1:1000; Abcam, ab246518) or RBM47 (1:1000; Abcam, ab167164). The following day, the sections were incubated with secondary antibodies and stained with diaminobenzidine. Subsequently, based on the intensity of staining, samples were graded on a scale from 0 (no staining) to 3 (intense staining). The proportion of cells displaying positive staining was then determined and ranged from 0 to 100%. The final IHC score was calculated by multiplying the intensity score with the percentage of positive cells, resulting in a score ranging from 0 to 300.

### Multiplexed immunofluorescence staining

Multiplex staining was performed using a TSA 2-color kit (D110071-50T, Yuanxibio) in accordance with the manufacturer’s guidelines. Following consecutive sectioning, the slides were incubated with primary antibodies against CD21 (1:100; Abcam, ab315160) and CD23 (1:100; Abcam, ab135386), followed by enzyme-labeled secondary antibodies (PV-6001 and PV-6002, ZSGB-BIO) and tyramide signal amplification (M-D110051, WiSee Biotechnology). Following each TSA operation, the slides were subjected to a microwave heat treatment. Subsequently, the nuclei were stained with 4′,6-diamidino-2-phenylindole (DAPI) (D1306, Thermo Fisher) after labeling with all aforementioned antigens. The stained slides were then scanned to generate multispectral images using a Pannoramic MIDI imaging system (3D HISTECH). These images were acquired for subsequent analysis using HALO Software (Indica Labs).

### Pathological evaluation of TLS

TLS was analyzed in CC tissues by detecting CD21 and CD23 using the tyramide signal amplification (TSA) approach according to the manufacturer’s protocol. IHC staining for CXCL13 and RBM47 in the TLS region was performed as previously described. IHC scores were obtained using HALO Software.

### Statistical analysis

Significant differences between the two groups were determined using either an unpaired or paired t-test. The chi-square test or Fisher’s exact test was employed to compare differences in categorical variables. Pearson’s correlation test was utilized for correlation analyses. Kaplan-Meier analysis and log-rank tests were deployed to analyze DFS and OS of patients with CC. Univariate and multivariate analyses were performed using Cox regression survival analyses. All statistical analyses were performed using GraphPad Prism V.8.0.0 (GraphPad Software). Differences were considered significant at *p < 0.05, **p < 0.01, or ***p < 0.001.

## Results

### Screening of immune cell module-associated genes

**Fig. 1:**
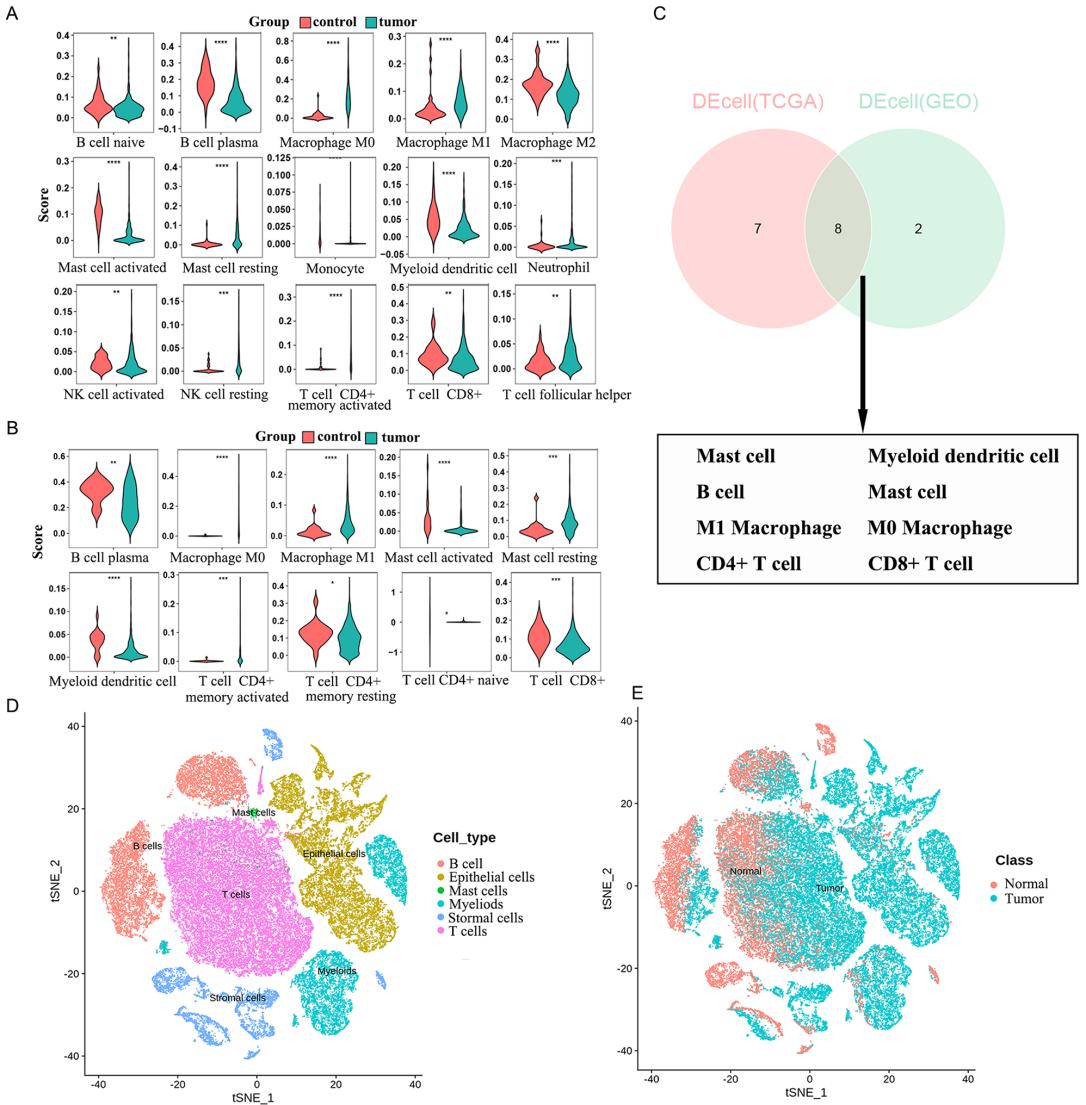
Screening of immune cell module-associated genes. (**A**) A total of 15 differential immune cells between the tumor group and control samples of TCGA-CC. (**B**) Ten differential immune infiltrating cells were in GSE39582. (**C**) Venn diagram analysis depicted the differentially immune cells between tumor group and control samples. (**D**) UMAP plot of six cell clusters. (**E**) Two-dimensional t-SNE visualization of six major cell types identified from normal and tumor samples

There were 15 differential immune cells between the tumor group and the control TCGA-CC samples (Fig. 1A and Supplementary Fig. S1A). Only 10 differential immune-infiltrating cells were found in GSE39582 (Fig. 1B and Supplementary Fig. S1B). Figure 1C displays eight shared differential immune cells in both datasets. Cluster analysis using WGCNA showed no outlier samples (Supplementary Fig. S1C). β = 7 was chosen to ensure network accuracy (Supplementary Fig. S1D). Modules with a correlation greater than 0.75 were merged to obtain 19 modules (Supplementary Fig. S1E). The purple and light green modules had a strong correlation with immune cells, and we chose these two modules as key modules (Supplementary Fig. S1F). Moreover, 493 genes associated with immune cells were obtained in the two key modules based on |GS|>0.2 and |MM|>0.5 screens (Supplementary Fig. S1G).

After quality control, 34,383 core cells were obtained (Supplementary Fig. S2A). After data normalization, 2000 highly variable genes were identified for subsequent analysis (Supplementary Fig. S2B). PCA analysis was performed on single-cell samples, and the data indicated no significant batch effects after integration (Supplementary Fig. S2C). Meanwhile, we selected the top 50 principal components for subsequent analysis (Supplementary Fig. S2D). The cell clusters were annotated according to the expression of marker genes in the clusters, and six-cell clusters were obtained (Fig. 1D). Moreover, Fig. 1E depicts the abundances of these immune cells between normal and tumor tissues. The marker genes in each cluster included IL7R, TFF3 and CD79A (Supplementary Fig. S2E). Based on the scRNA-seq data, DEGs were identified in the alloimmune cells of CC tumor and normal colon tissues.

### Construction and assessment of CC risk model

**Fig. 2:**
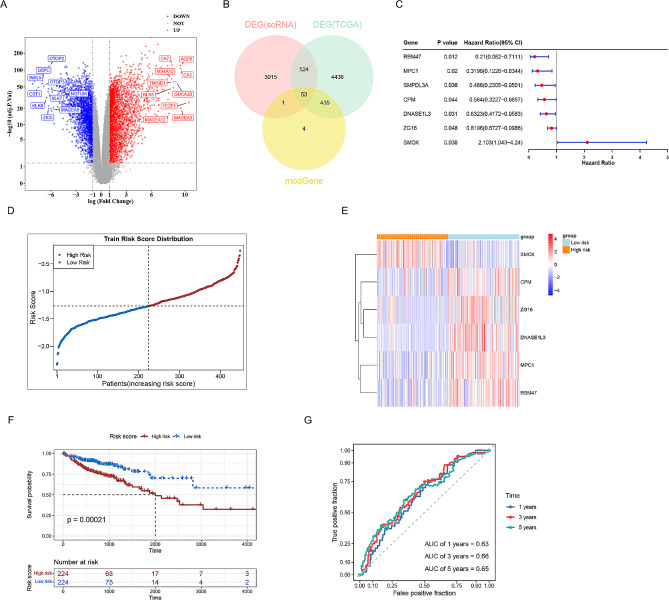
Identification of an immune signature by Cox proportional hazards model. (**A**) A total of 5448 differential genes shown by volcano maps: (red) up-regulated expressed genes, (blue) down-regulated genes, and (grey) non-differentially expressed genes. (**B**) Venn diagram of intersection genes of alloimmune cells-related DEGs, TCGA differentially expressed DEGs, and immune-related DEGs. (**C**) Univariate Cox regression revealed seven genes associated with prognosis. (**D**) The curve of risk score. (**E**) Heatmap shows the expression levels of signature genes. (**F**) The survival analysis of the two subgroups stratified based on the median of risk scores calculated by the risk model. (**G**) ROC curve analysis for the prognostic value of the prognostic model for different years

In the TCGA-CC dataset, there were 5448 DEGs, of which 2872 were up-regulated and 2576 were down-regulated in the tumor group compared with the normal group (Fig. 2A and Supplementary Fig. S3A). Using the Venn diagram, we identified 53 immune cell-related DEGs associated with CC, which were defined as candidate genes (Fig. 2B). Among the candidate genes, seven genes associated with CC prognosis were identified using univariate Cox analysis (Fig. 2C). After filtering by LASSO analysis, six characteristic genes (ZG16, MPC1, RBM47, SMOX, CPM and DNASE1L3) were obtained (Supplementary Fig. S3B). Based on the coefficients of the genes, we calculated the risk score as follows: risk score = ZG16 *(–0.04714834) + MPC1* (–0.58270763) + RBM47 * (–0.80011627) + SMOX * 0.34522437 + CPM *(–0.20786313) + DNASE1L3 * (–0.22272891). Accordingly, patients with TCGA-CC were divided into high-risk (*n* = 224) and low-risk categories (*n* = 224) based on the calculated median risk score (Fig. 2D and Supplementary Fig. S3C). The heatmap shows the differential expression of these six genes between the low- and high-risk groups (Fig. 2E). Interestingly, KM curves demonstrated that patients in the high-risk group had worse OS than those in the low-risk group (Fig. 2F). In addition, the ROC curves at 1, 3 and 5 years showed that the risk model had a better predictive power for patients (Fig. 2G). Furthermore, this risk model demonstrated good stability in the external dataset GSE41258 (Supplementary Fig. S4).

### Independent prognostic and nomogram model

**Fig. 3:**
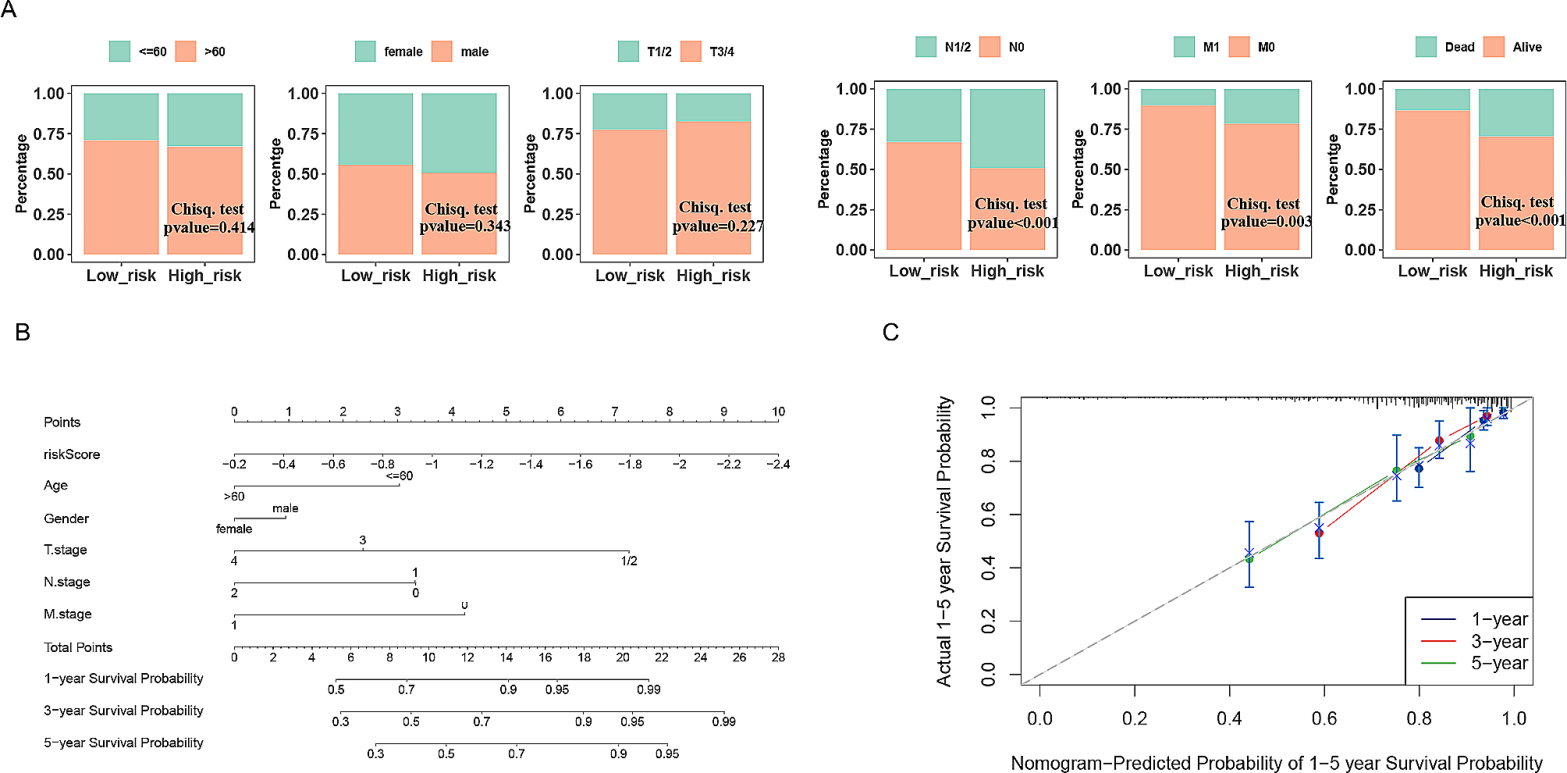
Establishment of the prognostic nomogram. (**A**) Correlation of risk group and clinical traits. (**B**) A predictive nomogram for predicting 1-, 3- and 5-year OS in CC patients. (**C**) The calibration plots for predicting 1-, 3- and 5-year OS

As presented in Fig. 3A, stage N, stage M and survival status were remarkably different between the high- and low-risk groups (*p* < 0.05). Nomograms constructed based on risk scores and clinical characteristics were good predictors of 1, 3 and 5-year survival in patients with CC (Fig. 3B). The C-index of the calibration curve was 0.79, indicating the feasibility of the model (Fig. 3C). Overall, the nomogram can serve as a reliable tool for predicting prognosis in patients with CC.

### RBM47 is down-regulated in CC, and this down-regulation is linked to poor patient survival

**Fig. 4:**
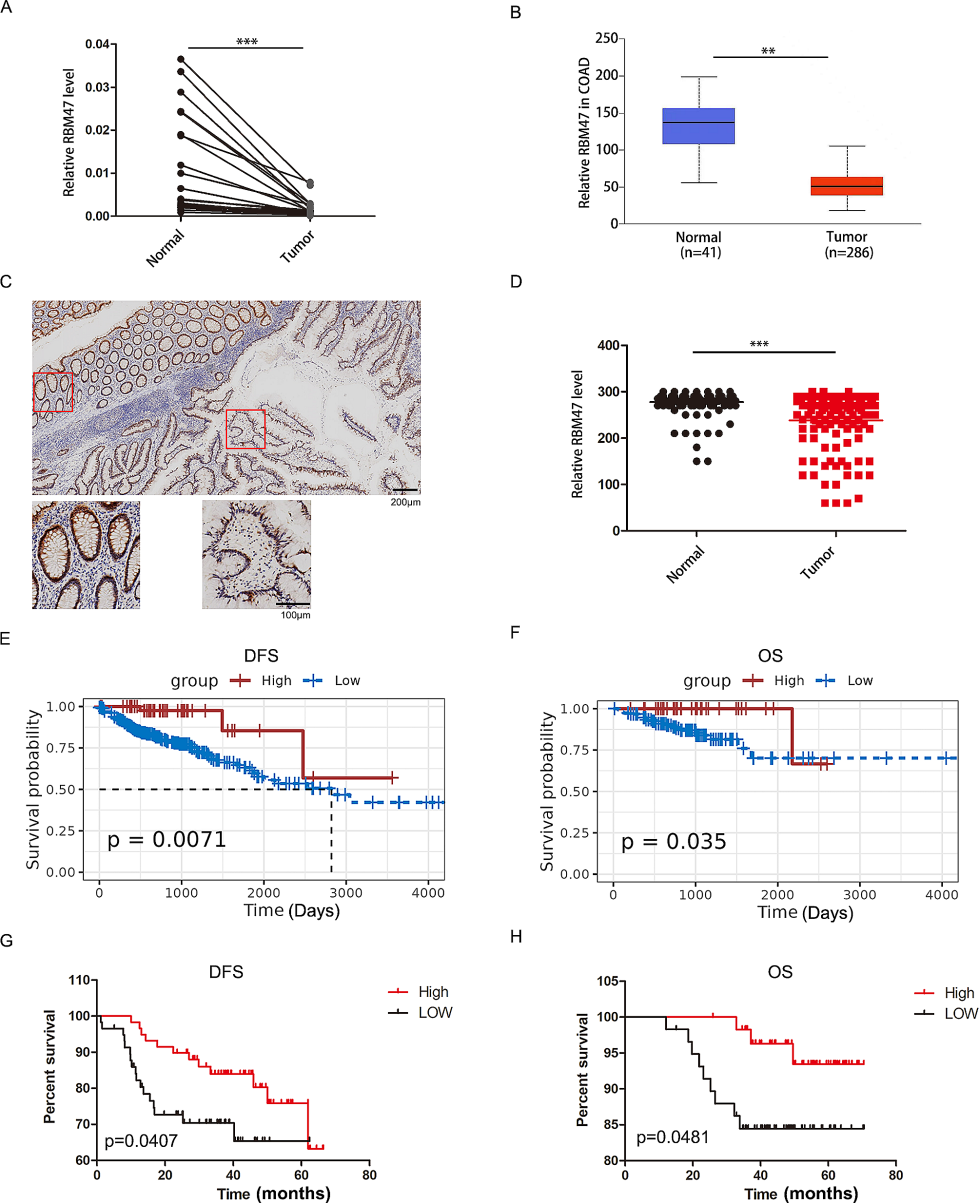
Upregulation of RBM47 expression in CC is correlated with good prognosis. (**A**) RBM47 expression levels were decreased in human CC samples compared with those in the paired noncancerous tissues (*n* = 20). (**B**) RBM47 mRNA expression level in COAD tissues from TCGA database. (**C-D**) Representative image of IHC staining of RBM47 in CC and paired noncancerous tissues. Scale bar: 100 μm. (**E-F**) Kaplan–Meier DFS and OS curve of CC patients correlated with RBM47 expression in TCGA dataset. (**G-H**) Kaplan–Meier DFS and OS curve of CC patients correlated with RBM47 expression in 118 patients with CC

Next, we preliminarily validated the expression levels of the six hub genes in the CC and adjacent normal tissues (*n* = 20). The clinical characteristics of the patients are listed in Table 1. Real-time quantitative PCR results disclosed that MPC1, ZG16, RBM47, CPM and DNASE1L3 were expressed at higher levels in adjacent normal tissues than in tumor tissues, and SMOX expression was higher in tumor tissues than in non-tumor tissues (Fig. 4A and Supplementary Fig. S5A). As RBM47 is the gene with the most significant change, we chose it for further intensive study. Furthermore, we validated RBM47 expression in TCGA database (COAD) (Fig. 4B). We next explored the relationship between RBM47 and the clinical features of CC. A total of 118 patients with CC were enrolled, and their clinical characteristics are summarized in Table 1. IHC results revealed that RBM47 expression was significantly reduced in CC samples (Fig. 4C, D). Furthermore, we explored a potential link between the expression level of RBM47 and MSI/MSS status of our cohort comprising 17 dMMR and 101 pMMR-colon cancers. The RBM47 level was positively correlated with dMMR (Table 1).

As presented in the KM survival curve, patients with CC in the high-RBM47 group had markedly longer DFS (Fig. 4E) and OS (Fig. 4F) rates than those in the low-RBM47 group. Consistently, our clinical samples verified that the high RBM47 group had better DFS (Fig. 4G) and OS (Fig. 4H). The factors associated with OS and DFS were evaluated using univariate and multivariate Cox regression models. RBM47 expression, gender, differentiation, T stage and N stage were found to correlate with the survival of patients with CC (Tables 2 and 3). Importantly, multivariate analysis demonstrated that RBM47 was an independent prognostic factor for worse OS and DFS among patients with CC (Tables 2 and 3). Through GSEA, we identified and predicted the possible biological functions of RBM47 in CC. An evident correlation was found between RBM47 expression and TNFA signaling, protein secrection and inflammatory response pathways (Supplementary Fig. S5B). Taken together, these results suggest an important regulatory role of RBM47 in CC.

**Table 2:** Univariate and Multivariate analysis for DFS of CC patients

Variables	Univariate analysis			Multivariate analysis		
	Hazard ratio	(95% CI)	*P*	Hazard ratio	HR (95%CI)	*P*
RBM47	0.41	0.30–0.55	< 0.0001	0.45	0.32–0.64	< 0.0001
Gender	0.34	0.14–0.84	0.0192	0.44	0.19–1.28	0.1434
Age	1.01	0.69–1.48	0.9480	-		-
Differentiation	0.44	0.23–0.86	0.0162	0.62	0.30–1.28	0.3706
T stage	2.76	1.25–6.09	0.0120	1.81	0.74–4.40	0.4535
N stage	1.81	1.06–3.06	0.0285	1.69	0.64–4.43	0.4925
AJCC stage	1.76	0.79–3.92	0.1658	0.64	0.13–3.15	0.8160
Location	1.33	0.64–2.78	0.4447	-		-
MMR status	1.85	0.56–6.12	0.3148	-		-
chemotherapy	0.63	0.25–1.56	0.3137	-		-

**Table 3:** Univariate and multivariate analysis for OS of CC patients

Variables	Univariate analysis			Multivariate analysis		
	Hazard ratio	(95% CI)	*P*	Hazard ratio	HR (95%CI)	*P*
RBM47	0.31	0.2–0.49	< .0001	0.33	0.20–0.54	< 0.0001
Gender	0.72	0.22–2.41	0.5983	-	-	-
Age	0.91	0.52–1.59	0.7350	-	-	-
Differentiation	0.23	0.08–0.71	0.0103	0.39	0.10–1.47	0.163
T stage	3.06	0.94–9.99	0.0644	3.96	0.93–16.9	0.064
N stage	1.73	0.78–3.82	0.1751	0.83	0.30–2.31	0.724
AJCC stage	2.03	0.55–7.55	0.2906	-	-	-
Location	2.13	0.64–7.08	0.2180	-	-	-
MMR status			0.9913	-	-	-
chemotherapy	0.36	0.11–1.21	0.0986	0.28	0.07–1.22	0.0906

### Immune characterizations analysis

**Fig. 5:**
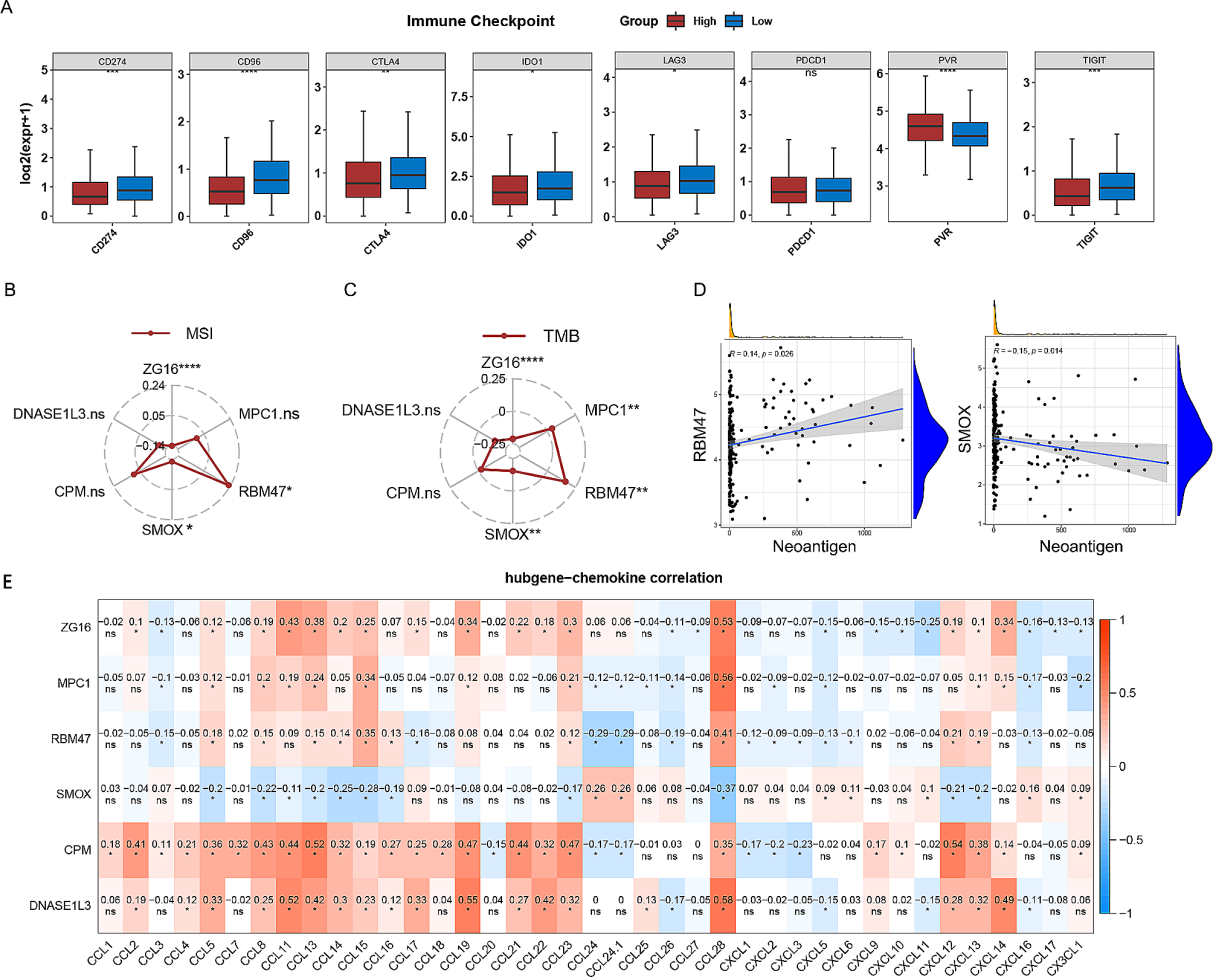
The relationship between hub gene expression and immune checkpoint genes, TMB, MSI, NEO and chemokines. (**A**) Different expressions of immune checkpoint genes in high- and low-risk groups. (**B**) The association between hub gene expression and MSI. (**C**) Association between hub gene expression and TMB. (**D**) The relationship between RBM47, SMOX expression and NEO. (**E**) Correlation between hub genes and chemokines

Recently, immune checkpoint inhibitors have been shown to play an important role in cancer treatment [31]. By analyzing the immune checkpoints, we found that with the exception of PDCD1, all seven immune checkpoints differed significantly between the high-risk and low-risk groups (*p* < 0.05) (Fig. 5A). Pearson’s correlation analysis was conducted between immune checkpoints that were markedly different in expression between low-risk and high-risk categories. Besides, the risk scores were significantly positively correlated with PVR and adversely correlated with CD274, CTLA4, CD96 and TIGIT (Supplementary Fig. S6A). MSI, TMB and NEOs are strongly linked to tumorigenesis and progression; therefore, we studied the relationships between hub gene expression and MSI, TMB and NEOs. RBM47 was positively associated with MSI, whereas ZG16 and SMOX were negatively correlated with MSI (Fig. 5B). ZG16, MPC1, RBM47 and SMOX were significantly linked to TMB (Fig. 5C). Similarly, RBM47 was markedly positively associated with NEOs, SMOX was significantly negatively correlated with NEOs (*p* < 0.05), and the other genes were not significantly linked to NEO (Fig. 5D and Supplementary Fig. S6B). In addition, we found that the hub genes were closely associated with chemokines (Fig. 5E). These results suggest that hub genes play an essential role in immunomodulatory processes in tumors.

#### Discovery of RBM47 expression in TLS

**Fig. 6:**
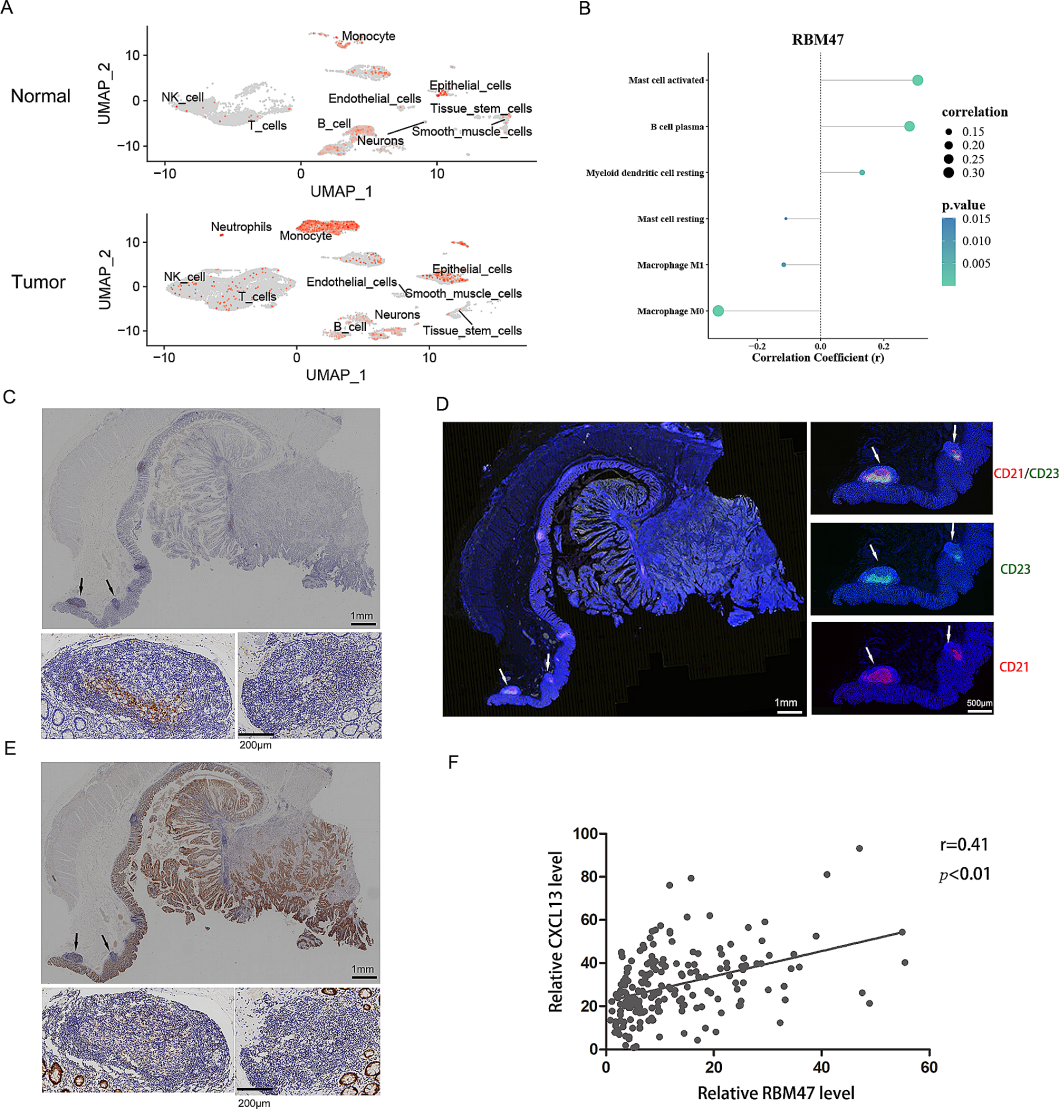
Discovery of RBM47 expression in TLS. (**A**) RBM47 expressed specifically in epithelial cells and monocytes clustered from integrated single-cell RNA sequence data. (**B**) Correlation coefficient plot of RBM47 and immune-infiltrating cells. (**C**) Representative TLS in tissues stained by H&E and CXCL13 by IHC. (**D**) Representative image of IHC staining of RBM47 in CC tissues. Arrows indicate TLS. (**E**) Multiplex immunofluorescence assay of CD23 (green) and CD21 (red). (**F**) The correlation between RBM47 and CXCL13 in the TLS region

At the single-cell level, RBM47 was mainly expressed in epithelial cells and monocytes (Fig. 6A). As reported, monocytes are considered the definitive precursors of DCs [32]. RBM47 expression was significantly positively correlated with myeloid DCs (Fig. 6B). Recent studies have reported that follicular DCs are a major source of CXCL13 [33]. The TLS is formed by B cells that are recruited to tumors by CXCL13 [34]. In line with previous observations, CXCL13 was constitutively expressed in TLS (Fig. 6C). Analysis of the correlation between characteristic genes and chemokines demonstrated that CXCL13 was significantly positive with RBM47 (Fig. 5E). As reported, TLS maturation is an important parameter of tumor immune contexture and bears significant prognostic and potential predictive value in CRC [35]. Multiplexed immunofluorescence staining was performed to validate the presence of mature TLS in the CC tissues (Fig. 6D). Accordingly, IHC analysis revealed that RBM47 was preferentially presented within TLS in interstitial regions (Fig. 6E). Surprisingly, we observed that RBM47 was positively correlated with CXCL13 in the TLS region (Fig. 6F). In general, RBM47 may orchestrate TLS formation by regulating CXCL13 levels.

## Discussion

Progress in identifying early diagnostic biomarkers and therapeutic targets for CC has been comparatively sluggish, impeding the achievement of favorable clinical outcomes [36]. Accumulating evidence indicates that the tumor immune contexture comprising the spatial arrangement, abundance, and functional orientation of immune cells infiltrating the tumor significantly influences the clinical prognosis of cancer patients [37].

In the present study, immune-related differential genes were screened using bulk and scRNA sequencing data. Subsequently, six prognostic DEGs were identified using survival and immune analyses. Additionally, a nomogram model was constructed and validated to predict the prognosis of patients with CC. Verified by clinical samples, we chose RBM47 for further intensive studies. RBM47 is an RNA-binding protein that predominantly binds to introns and 3′-UTRs of its target mRNAs, thereby regulating their stability [13]. In support of our findings, RBM47 could inhibit CRC cell proliferation, invasion and migration by targeting the PTEN/PI3K/AKT signaling pathway [38]. Furthermore, RBM47 silencing was highly associated with CRC progression and epithelial-mesenchymal transition(EMT) [17]. Previous studies have indicated that RBM47 has demonstrated inhibitory effects on tumor progression in gastric [14], hepatocellular [16] and breast cancers [15]. Conversely, other studies have suggested that RBM47 may exhibit oncogenic properties in nasopharyngeal carcinoma via its role as a DNA/RNA-binding protein [39]. It is hypothesized that the dual nature of RBM47’s impact on tumorigenesis may be influenced by factors such as tumor heterogeneity, pathological subtypes, or molecular mechanisms, leading to disease-specific outcomes.

In our study, using IHC staining, we found that RBM47 expression was significantly lower in CC tissues than in normal colon tissues. Subsequently, increased RBM47 levels in cancer tissue samples were strongly linked to the clinicopathological characteristics of CC and served as independent predictors of extended OS and DFS. These results were consistent with the TCGA-CC dataset. IHC results demonstrated that RBM47 was down-regulated in CC. Accordingly, an increase in RBM47 was significantly associated with good prognosis in patients with CC in multivariate and univariate analyses.

MSI serves as a crucial predictor of tumor initiation and progression [40]. TMB influences the likelihood of generating immunogenic peptides, thereby affecting the prognosis of CRC [41]. Mismatch repair deficiency in CRC can lead to MSI and an elevated count of NEOs compared with microsatellite stable (MSS) tumors [42]. Our study demonstrated a positive correlation between RBM47 expression and the presence of TMB, MSI and NEOs in CC. Consequently, our findings suggested that RBM47 expression may serve as a valuable indicator for predicting responses to immune regulatory processes.

Generally, tumor development and progression are dependent on the TME, which consists of various non-malignant cell types and extracellular components [4]. Our study identified RBM47 as being enriched in monocytes and epithelial cells, with a positive correlation between RBM47 expression and multiple immune cell types, particularly mast cells, DCs and B cells, indicating a complex infiltration pattern. Prior studies have demonstrated the significance of RBM47 in the post-transcriptional regulation of IL-10, thereby enhancing the regulatory capabilities of B cells and implicating RBM47 in cancer immunity modulation [43]. IHC results further revealed RBM47 expression in various cell types, including immune and intestinal epithelial cells.

Chemokines are recognized for their capacity to induce cell migration and are essential for facilitating immune cell infiltration [44]. The present study identified a notable positive association between RBM47 and CXCL13 expression using bioinformatics analysis. Subsequently, IHC was employed to confirm the positive correlation between CXCL13 and RBM47 expression. Accumulating evidence indicates that follicular DCs and Tfh cells are significant producers of CXCL13, which in turn attracts CXCR5-expressing immune cells and promotes TLS formation [45]. This process enhances the immune response and activates the cytotoxic effects of immune cells against tumors. Tumor-associated TLSs exhibit lymph node-like characteristics, such as a T-cell zone containing DCs and a germinal center with follicular DCs and proliferating B cells, suggesting a crucial role of these lymphoid structures in regulating adaptive anti-tumor immunity [46].

The presence of TLS surrounding tumors has garnered heightened interest as an immune barrier, and meta-analyses have demonstrated that elevated TLS expression in solid tumors is correlated with extended overall survival, reduced risk of tumor recurrence, smaller tumor size, increased tumor-infiltrating lymphocytes (TILs), lower tumor grade and lower N stage [34]. Furthermore, numerous studies have demonstrated a strong correlation between increased TLS density and improved prognosis in patients with CRC [12]. It has been hypothesized that RBM47 may play a crucial role in the organization of TLS by modulating CXCL13 levels. Overall, our findings suggest that RBM47 could serve as a potential biomarker for the early detection of CRC, as well as a promising therapeutic target for the prognostic assessment and treatment of patients with this disease.

The current research offers robust evidence supporting the predictive significance of RBM47 in clinical prognosis and its positive association with CXCL13 within the TLS region. However, this study has certain limitations. Although the relationship between RBM47 and CXCL13 has been extensively studied, the regulatory mechanisms governing their production remain unclear. Furthermore, understanding the specific conditions conducive to TLS formation is crucial to identify potential therapeutic targets. The co-localization of RBM47 and TLSs may provide insight into the formation of these structures.

## Conclusion

Generally, our research revealed the direct role of RBM47 in suppressing CC and modulating CXCL13 within TLS, emphasizing the significance of RBM47 in promoting anti-tumor immune responses and impeding tumor advancement. Our results also propose that reinstating RBM47 could serve as a promising strategy for anti-cancer treatment.

### Electronic supplementary material

Below is the link to the electronic supplementary material.

Supplementary Material 1

Supplementary Material 2

Supplementary Material 3

Supplementary Material 4

Supplementary Material 5

Supplementary Material 6

Supplementary Material 7

Supplementary Material 8

## Data Availability

The datasets used and analysed during the current study are available from the corresponding author on reasonable request. Original datasets are available in a publicly accessible repository: The original contributions presented in the study are publicly available. These data can be found in TCGA (https://portal.gdc.cancer.gov/) and GEO database (https://www.ncbi.nlm.nih.gov/).

## Abbreviations

CC: Colon cancer
WGCNA: Leighted gene co-expression network analysis
LASSO: Least absolute shrinkage and selection operator
KM: Kaplan-Meier
TMB: Tumor mutation burden
MSI: Microsatellite instability
MSS: Microsatellite stable
NEO: Neoantigen
DFS: Disease-free survival
OS: Overall survival
TLS: Tertiary lymphoid structure
TME: Tumor microenvironment
DEGs: Differentially expressed genes
EMT: Epithelial-mesenchymal transition
H&E: Hematoxylin and eosin
IHC: Immunohistochemistry
