# Supplementary material for: Constructing a prognostic model for colon cancer: insights from immunity-related genes
Source: BMC Cancer. 2024 Jun 24;24:758. doi: 10.1186/s12885-024-12507-z (PMC11197172; doi:10.1186/s12885-024-12507-z)
Supplement: Supplementary file 1 — Supplementary Material 1 [file 12885_2024_12507_MOESM1_ESM.docx]

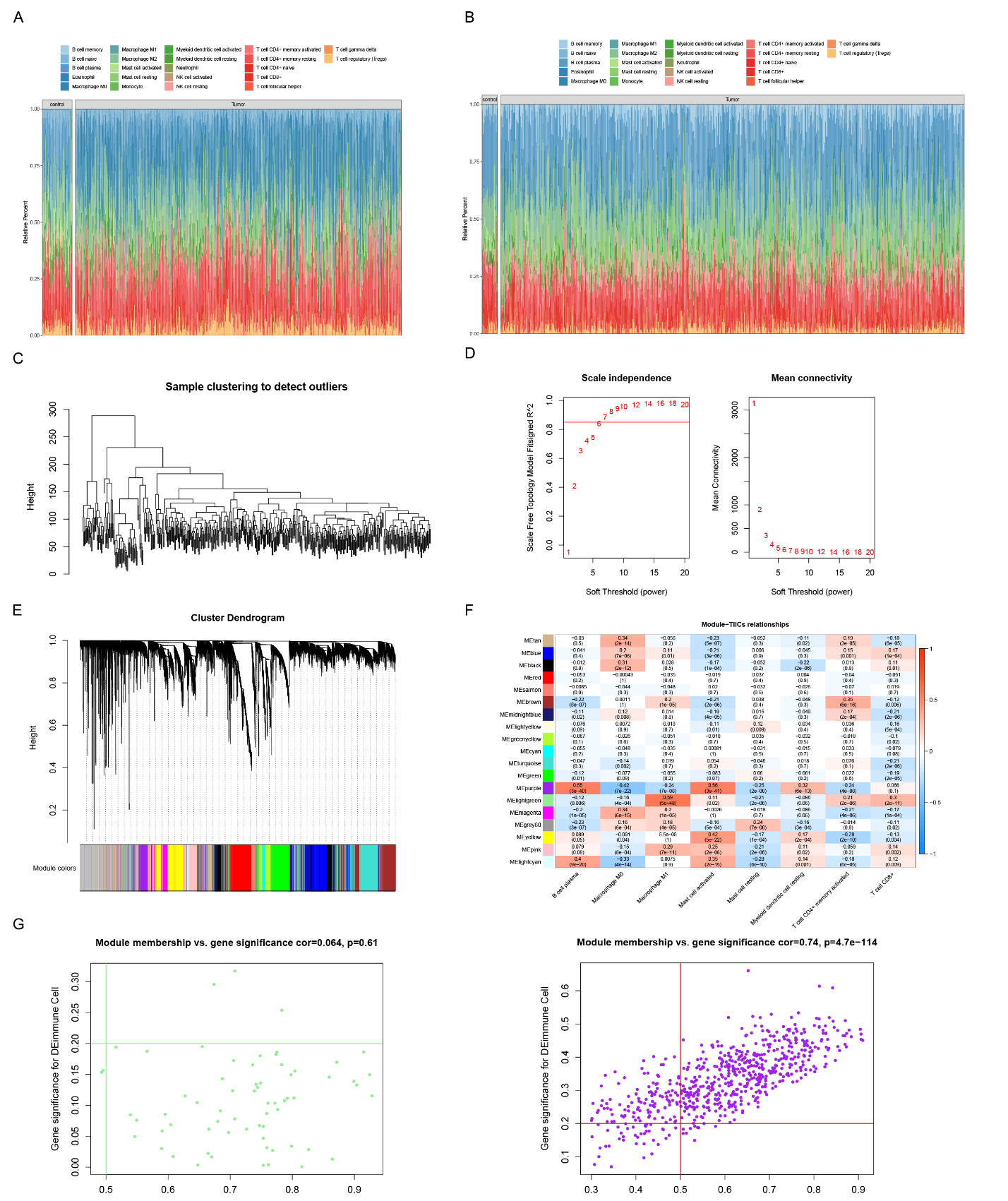


**Supplementary Figure S1.** Screening of immune cell module-associated genes. **(A)** Differential immune cells between the tumor and control samples of TCGA-CC. **(B)** Differential immune-infiltrating cells were found in the GSE39582 dataset. **(C)** Cluster dendrogram of CC samples from TCGA. **(D)** Left: analysis of the scale-free fit index for various soft-thresholding powers (Î ² =7); right: analysis of the mean connectivity for various soft-thresholding powers (β=7). **(E)** Dendrogram of the gene cluster. Each module is distinguished by different colors. **(F)** Heatmap of the correlation between modules and phenotypic traits of CC.  **（G）**  A highly significant correlation between gene significance and module membership.
