# Supplementary material for: Constructing a prognostic model for colon cancer: insights from immunity-related genes
Source: BMC Cancer. 2024 Jun 24;24:758. doi: 10.1186/s12885-024-12507-z (PMC11197172; doi:10.1186/s12885-024-12507-z)
Supplement: Supplementary file 2 — Supplementary Material 2 [file 12885_2024_12507_MOESM2_ESM.docx]

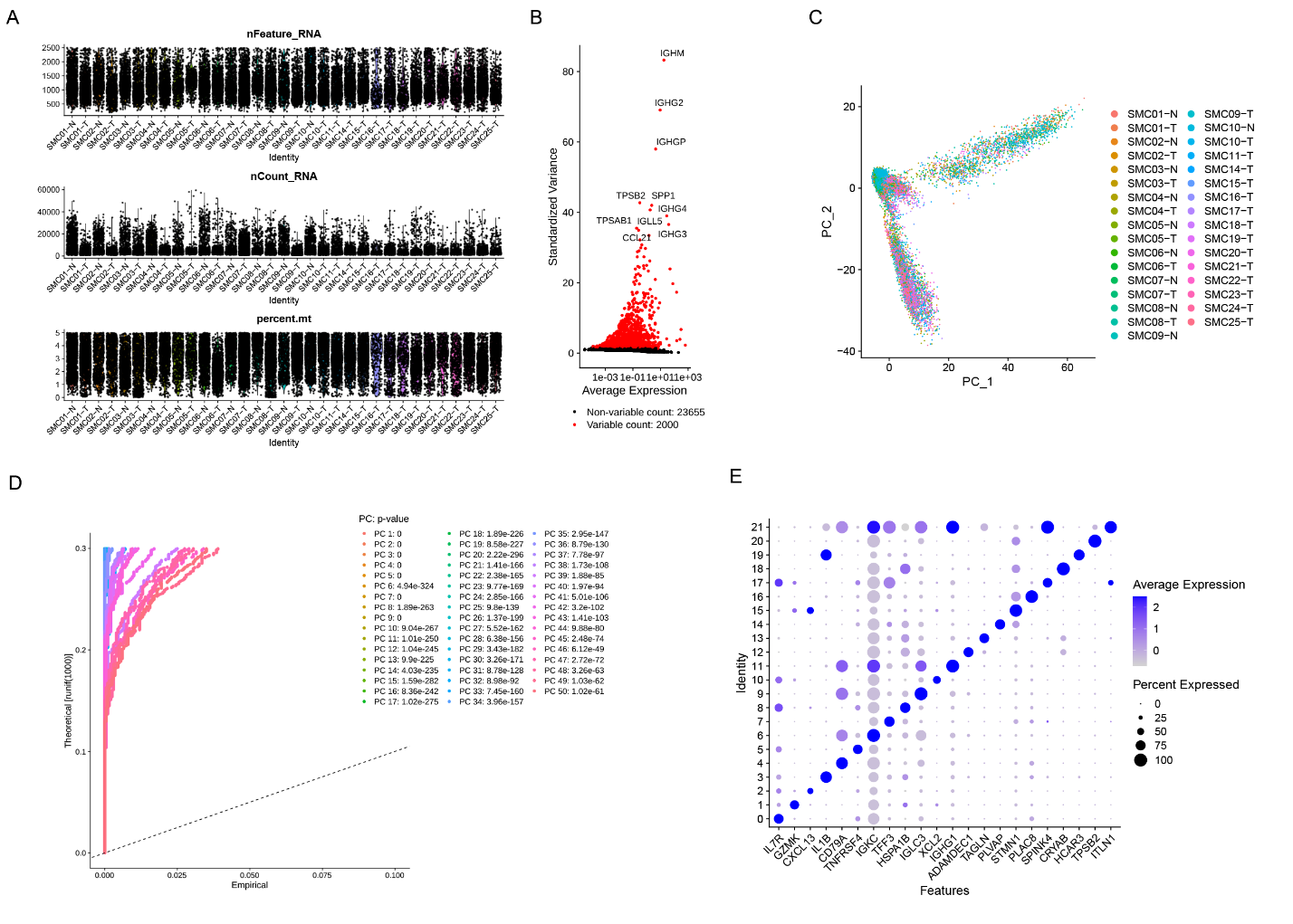


**Supplementary Figure S2.** Identification of DEGs in alloimmune cells between CC tumors and normal colon tissues. **(A)**  A total of 34,383 core cells were obtained. **(B)** Top 2,000 genes with large coefficients of variation among cells. **(C)** PCA analysis was performed on single-cell samples. **(D)** Top 50 principal components. **(E)** Marker genes in each cluster.
