# Supplementary material for: Constructing a prognostic model for colon cancer: insights from immunity-related genes
Source: BMC Cancer. 2024 Jun 24;24:758. doi: 10.1186/s12885-024-12507-z (PMC11197172; doi:10.1186/s12885-024-12507-z)
Supplement: Supplementary file 3 — Supplementary Material 3 [file 12885_2024_12507_MOESM3_ESM.docx]

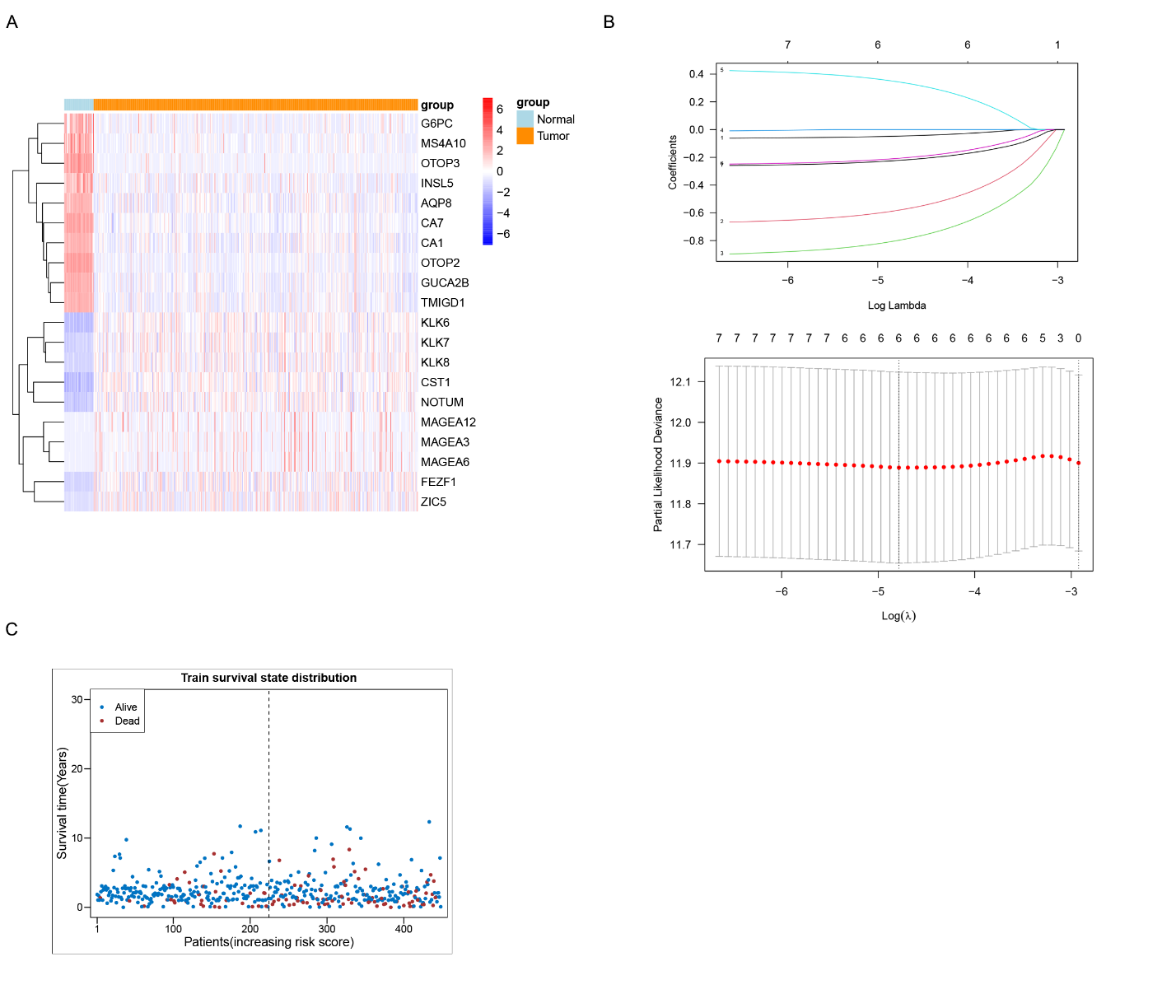


**Supplementary Figure S3.** Construction and assessment of CC risk model. **(A)**  Heatmap displaying the expression levels of the signature genes. **(B)** Process of establishing a risk signature using six glycosyltransferase genes. Coefficients were calculated using multivariate Cox regression with LASSO. **(C)** Distribution of survival status in patients with CC in TCGA.
