# Supplementary material for: Constructing a prognostic model for colon cancer: insights from immunity-related genes
Source: BMC Cancer. 2024 Jun 24;24:758. doi: 10.1186/s12885-024-12507-z (PMC11197172; doi:10.1186/s12885-024-12507-z)
Supplement: Supplementary file 4 — Supplementary Material 4 [file 12885_2024_12507_MOESM4_ESM.docx]

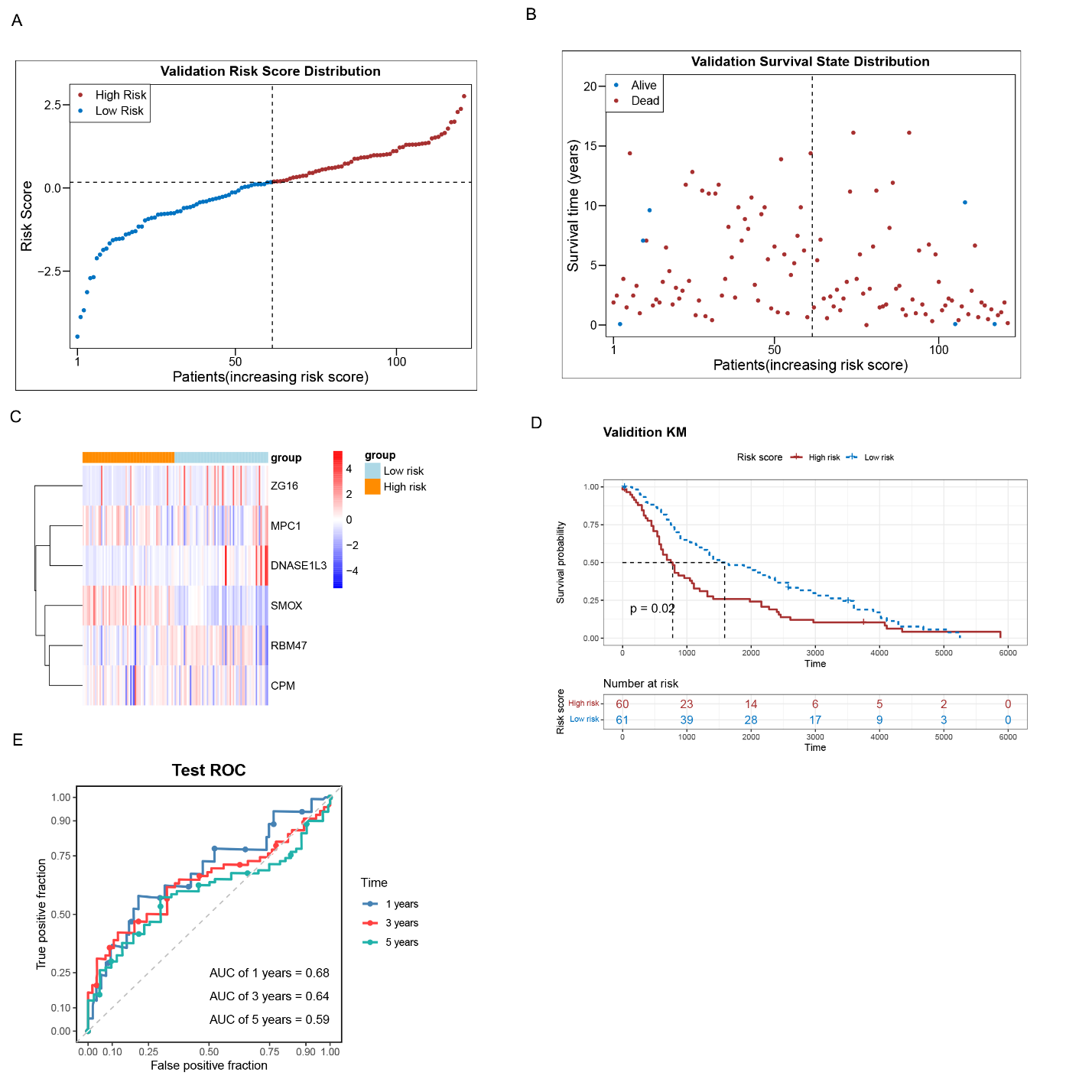


**Supplementary Figure S4.** Risk model conformed to GSE41258. **(A)** Distribution of immune risk scores in patients with CC.  **(B)** Survival status distribution of patients with CC. **(C)** Heatmap indicating the expression levels of the signature genes. **(D)** Survival analysis of the two subgroups stratified based on the median risk scores calculated by the risk model. **(E)** ROC curve analysis of the prognostic value of the prognostic model for different years.
