# Supplementary material for: Constructing a prognostic model for colon cancer: insights from immunity-related genes
Source: BMC Cancer. 2024 Jun 24;24:758. doi: 10.1186/s12885-024-12507-z (PMC11197172; doi:10.1186/s12885-024-12507-z)
Supplement: Supplementary file 5 — Supplementary Material 5 [file 12885_2024_12507_MOESM5_ESM.docx]

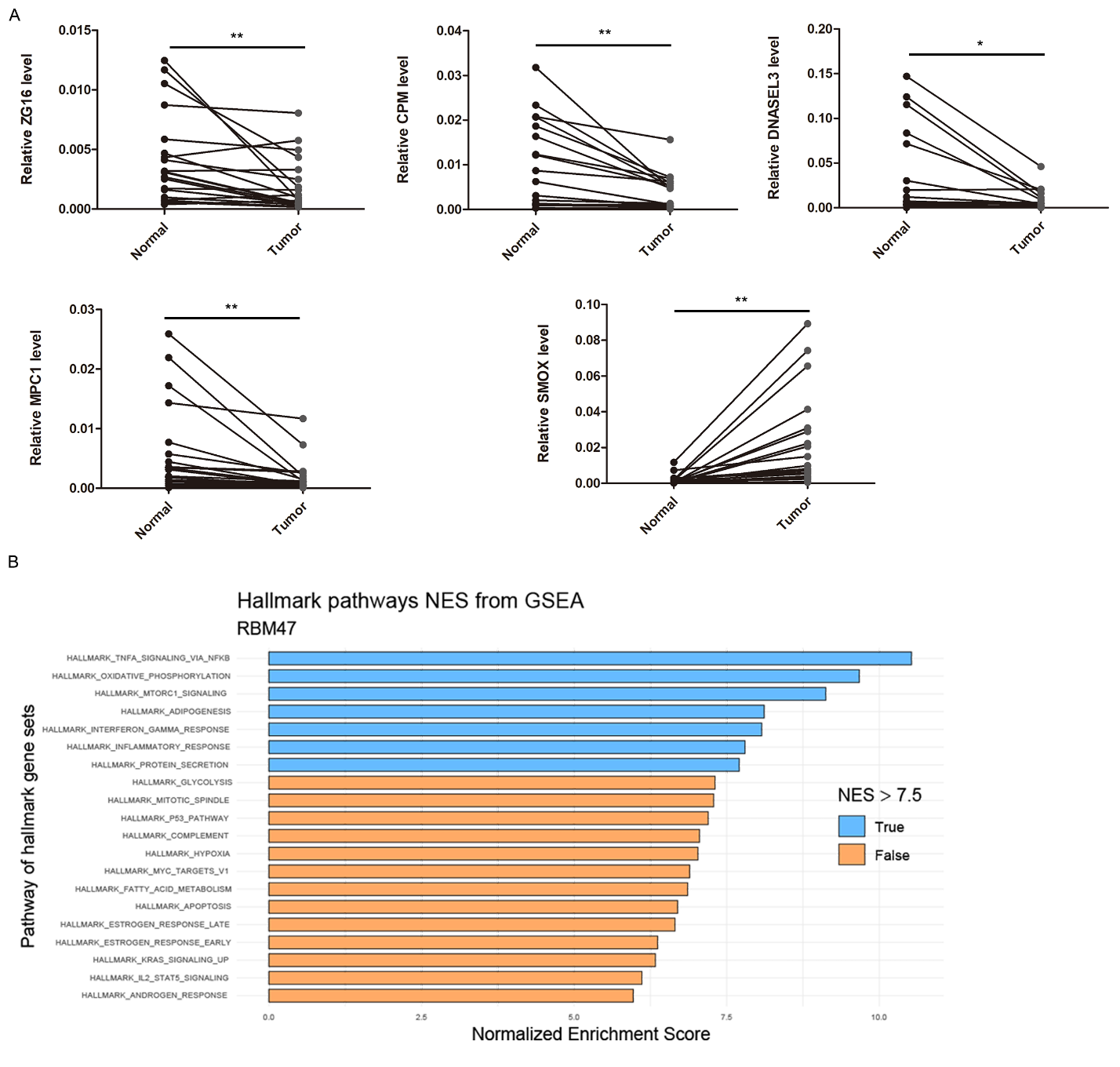


**Supplementary Figure S5.** **(A)** Five hub genes (ZG16, CPM, DNASE1L3, MPC1 and DNASE1L3) were identified in CC samples and adjacent normal tissues (n=20). **(B)** GSEA analysis showing enrichment pathways in RBM47-high samples.
