# Supplementary material for: Constructing a prognostic model for colon cancer: insights from immunity-related genes
Source: BMC Cancer. 2024 Jun 24;24:758. doi: 10.1186/s12885-024-12507-z (PMC11197172; doi:10.1186/s12885-024-12507-z)
Supplement: Supplementary file 6 — Supplementary Material 6 [file 12885_2024_12507_MOESM6_ESM.docx]

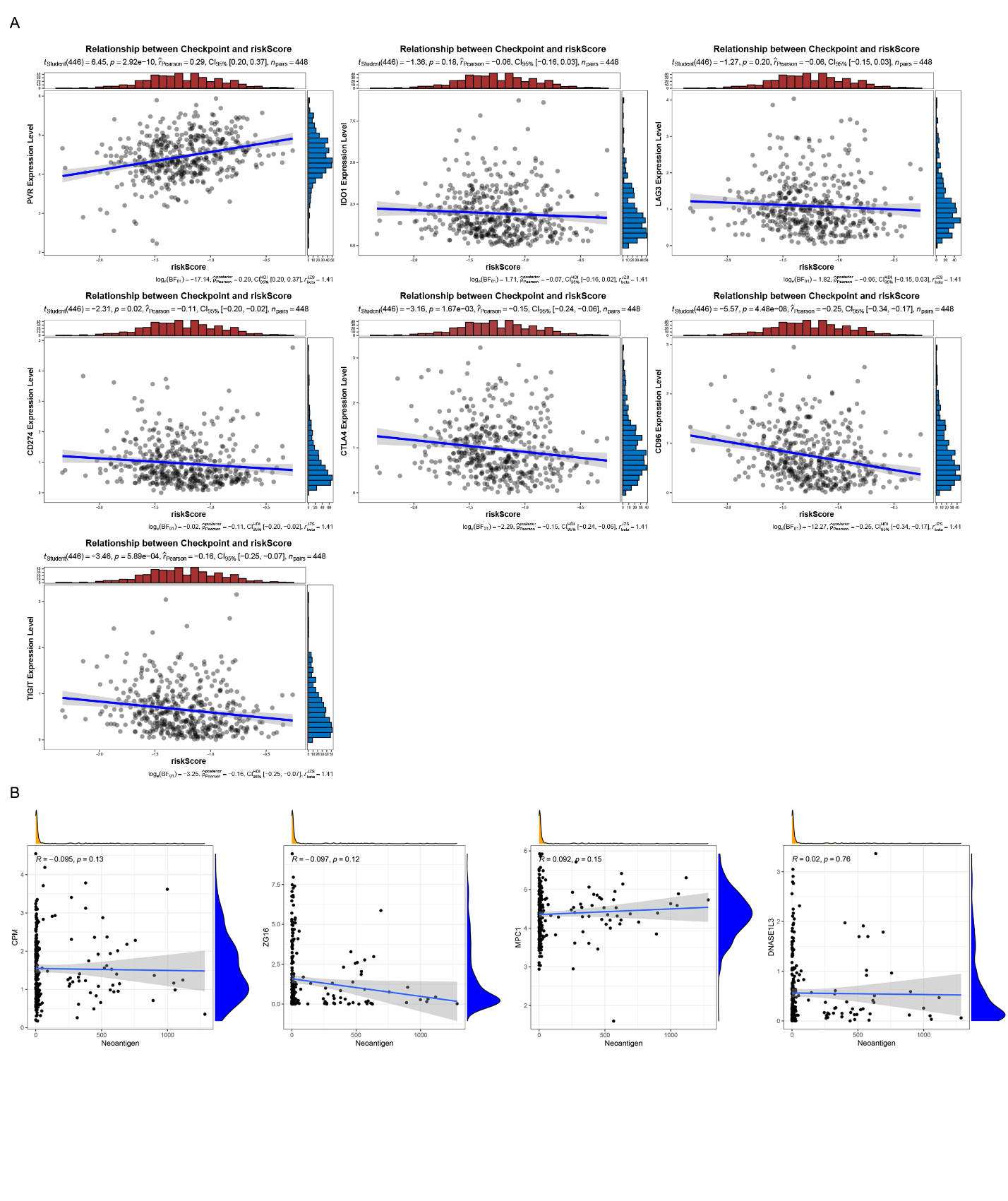


**Supplementary Figure S6.** **(A)** Pearson’s correlation analysis between immune checkpoints was expressed markedly differently in the low- and high-risk categories. **(B)** Association between the four hub genes and NEOs.
