# Supplementary material for: Constructing a prognostic model for colon cancer: insights from immunity-related genes
Source: BMC Cancer. 2024 Jun 24;24:758. doi: 10.1186/s12885-024-12507-z (PMC11197172; doi:10.1186/s12885-024-12507-z)
Supplement: Supplementary file 8 — Supplementary Material 8 [file 12885_2024_12507_MOESM8_ESM.docx]

**Supplementary Table S2.** Primer sequences used in this research.

| **Gene** | **Sequence (5'-3')** |
| --- | --- |
| RBM47-F | ATCAGCAATCCTTGGCTCAC |
| RBM47-R | CCTTGGGATTCCTCTGTTCA |
| UPF1-F | AAGGTATGGCGTCATCATTGTGG |
| UPF1-R | CCGTGGCTTGCTGAACTGC |
| UPF1 (for ChIP)-F | ATGGTCTCGATCTCCTG |
| UPF1 (for ChIP)-R | AAAGCTGCTTGGAACTC |
| GAPDH-F | GGTATCGTGGAAGGACTCAT |
| GAPDH-R | CCTTGCCCACAGCCTTG |
|  |  |
